# Supplementary material for: Factorized visual representations in the primate visual system and deep neural networks
Source: eLife. 2024 Jul 5;13:RP91685. doi: 10.7554/eLife.91685 (PMC11226229; doi:10.7554/eLife.91685)
Supplement: Supplementary file 1. — (a) Table of datasets used for measuring similarity of models to the brain. Datasets from both macaque and human high-level visual cortex as well as high-level visual behavior were collated for testing the brainlikeness of computational models. For neural and fMRI datasets, the features in the model were used to predict the image-by-image response pattern of each neuron or voxel. For behavior datasets, the performance of linear decoders built atop model representations were compared to performance per image of macaques and humans. (b) Table of models tested. For each model, we measured representational factorization and invariance in each of the final five layers of the model as well as evaluating their brainlikeness using the datasets in (a). [file elife-91685-supp1.docx]

### Supplementary File 1

| **Dataset** | **Key** | **#neurons, voxels** | **#subj** | **Image Stimuli** | **#images** |
| --- | --- | --- | --- | --- | --- |
| [DiCarlo-](https://www.jneurosci.org/content/35/39/13402.long)Majaj-Hong 2015^1^  Macaque V4 multi-unit activity | E1 | 128 | 2 | 6^o^, grayscale, synthetic | 5760 |
| [DiCarlo](https://www.jneurosci.org/content/35/39/13402.long)-Majaj-Hong 2015^1^  Macaque IT multi-unit activity | E1 | 168 | 2 | 6^o^, grayscale, synthetic | 5760 |
| [DiCarlo-Rust](https://www.jneurosci.org/content/32/30/10170.long) 2012^2^  Macaque V4 single neuron | E2 | 143 | 2 | 5^o^, grayscale, natural | 300 |
| [DiCarlo-Rust](https://www.jneurosci.org/content/32/30/10170.long) 2012^2^  Macaque IT single neuron | E2 | 142 | 2 | 5^o^, grayscale, natural | 300 |
| [DiCarlo-](https://www.jneurosci.org/content/38/33/7255.long)Rajalingham-Issa 2018^3^  Macaque behavior  Image-level classification | I1 | N/A | 5 | 6-8^o^, grayscale, synthetic | 240 |
| [DiCarlo-](https://www.jneurosci.org/content/38/33/7255.long)Rajalingham-Issa 2018^3^  Macaque behavior Image x class confusion matrix | I2 | N/A | 5 | 6-8^o^, grayscale, synthetic | 240 |
| [Gallant-Kay 2008](https://www.nature.com/articles/nature06713)^4^  Human V4 fMRI  ([dataset](http://crcns.org/data-sets/vc/vim-1/about-vim-1)) | F1 | 2,557 | 2 | 20^o^, grayscale, natural | 1870 |
| [Gallant-Kay 2008](https://www.nature.com/articles/nature06713)^4^  Human HVC fMRI ([dataset](http://crcns.org/data-sets/vc/vim-1/about-vim-1)) | F1 | 1,286 | 2 | 20^o^, grayscale, natural | 1870 |
| [Horikawa-Kamitani 2019](https://journals.plos.org/ploscompbiol/article?id=10.1371/journal.pcbi.1006633)^5^  Human V4 fMRI  ([dataset](https://openneuro.org/datasets/ds001506/versions/1.3.1)) | F2 | 3,377 | 3 | 12^o^, color, natural | 1250 |
| [Horikawa-Kamitani 2019](https://journals.plos.org/ploscompbiol/article?id=10.1371/journal.pcbi.1006633)^5^  Human HVC fMRI  ([dataset](https://openneuro.org/datasets/ds001506/versions/1.3.1)) | F2 | 14,465 | 3 | 12^o^, color, natural | 1250 |
| [DiCarlo-](https://www.jneurosci.org/content/38/33/7255.long)Rajalingham-Issa 2018^3^  Human behavior  Image-level classification | I1 | N/A | 1472 | 6-8^o^, grayscale, synthetic | 240 |
| [DiCarlo-](https://www.jneurosci.org/content/38/33/7255.long)Rajalingham-Issa 2018^3^  Human behavior  Image x class confusion matrix | I2 | N/A | 1472 | 6-8^o^, grayscale, synthetic | 240 |

**Supplementary file 1a. Table of datasets used for measuring similarity of models to the brain.** Datasets from both macaque and human high-level visual cortex as well as high-level visual behavior were collated for testing the brainlikeness of computational models. For neural and fMRI datasets, the features in the model were used to predict the image-by-image response pattern of each neuron or voxel. For behavior datasets, the performance of linear decoders built atop model representations were compared to performance per image of macaques and humans.

| **Model** | **Architecture** | **Loss Function** | **Customization** |
| --- | --- | --- | --- |
| SimCLR^6^ | ResNet-50 | Self-supervised (contrastive) | -------- |
| SimCLR^6^ | ResNet-50 | Self-supervised (contrastive) | 2x wide |
| SimCLR^6^ | ResNet-152 | Self-supervised (contrastive) | 2x wide |
| SimCLR^6^ | ResNet-50 | Self-supervised (contrastive) | no projection head |
| SimCLR^6^ | ResNet-50 | Self-supervised (contrastive) | only crop augmentations |
| SimCLR^6^ | ResNet-50 | Self-supervised (contrastive) | only crop augmentations, temperature 0.2 |
| SimCLR^6^ | ResNet-50 | Self-supervised (contrastive) | only crop augmentations, temperature 0.05 |
| SimCLR^6^ | ResNet-50 | Self-supervised (contrastive) | only crop and blur augmentations |
| SimCLR^6^ | ResNet-50 | Self-supervised (contrastive) | only crop and non-hue color jitter augmentations |
| SimCLR^6^ | ResNet-50 | Self-supervised (contrastive) | only crop, blur, and non-hue color jitter augmentations |
| MOCO^7^ | ResNet-50 | Self-supervised (contrastive) | -------- |
| MOCO v2^8^ | ResNet-50 | Self-supervised (contrastive) | -------- |
| MOCO v2^8^ | ResNet-50 | Self-supervised (contrastive) | only crop augmentations |
| MOCO v2^8^ | ResNet-50 | Self-supervised (contrastive) | only crop, color jitter, and grayscale augmentations |
| MOCO v2^8^ | ResNet-50 | Self-supervised (contrastive) | only crop augmentations, all image inputs preprocessed to grayscale |
| MOCO v2^8^ | ResNext-50 | Self-supervised (contrastive) | -------- |
| MOCO v2^8^ | ResNet-18 | Self-supervised (contrastive) | -------- |
| Instance discrimination^9^ | ResNet-50 | Self-supervised (image discrimination) | -------- |
| InfoMin^10^ | ResNet-50 | Self-supervised (contrastive) | -------- |
| InfoMin^10^ | ResNext-101 | Self-supervised (contrastive) | -------- |
| InfoMin^10^ | ResNext-152 | Self-supervised (contrastive) | -------- |
| SwAV^11^ | ResNet-50 | Self-supervised (cluster) | -------- |
| Deep clustering v2^12^ | ResNet-50 | Self-supervised (cluster) | -------- |
| BYOL^13^ | ResNet-50 | Self-supervised (no negative examples) | -------- |
| BYOL^13^ | ResNet-50 | Self-supervised (no negative examples) | only crop augmentations during training |
| BYOL^13^ | ResNet-50 | Self-supervised (no negative examples) | only crop and blur augmentations |
| BYOL^13^ | ResNet-50 | Self-supervised (no negative examples) | without color jitter augmentation |
| BYOL^13^ | ResNet-50 | Self-supervised (no negative examples) | without grayscale augmentation |
| BYOL^13^ | ResNet-50 | Self-supervised (no negative examples) | batch size 64 |
| BYOL^13^ | ResNet-50 | Self-supervised (no negative examples) | batch size 512 |
| Relative patch location^14^ | ResNet-50 | Auxiliary task (determine relative positions of image patches) | -------- |
| Rotation prediction^14^ | ResNet-50 | Auxiliary task (infer rotations that were applied given a set of images) | -------- |
| Colorization^15^ | ResNet-50 | Auxiliary task: (colorize grayscale images) | -------- |
| Jigsaw puzzle^16^ | ResNet-50 | Auxiliary task: (determine relative positions of image patches) | -------- |
| Big BiGAN^17^ | ResNet-50 | Auxiliary task (autoencoder objective with reconstruction error parameterized using a neural network discriminator) | -------- |
| ResNet^18^ | ResNet-50 | Supervised (classification) | -------- |
| ResNet^18^ | ResNet-50 | Supervised (classification) | MOCO data augmentations used during training |
| ResNet^18^ | ResNet-50 | Supervised (classification) | no data augmentation used during training |
| ResNet^18^ | ResNet-18 | Supervised (classification) | -------- |
| ResNet^18^ | ResNet-101 | Supervised (classification) | -------- |
| Wide ResNet^19^ | ResNet-50 | Supervised (classification) | -------- |
| AlexNet^20^ | AlexNet | Supervised (classification) | -------- |
| GoogLeNet^21^ | GoogLeNet | Supervised (classification) | -------- |
| Inception-v3^22^ | Inception-v3 | Supervised (classification) | -------- |
| DenseNet^23^ | DenseNet-169 | Supervised (classification) | -------- |
| DenseNet^23^ | DenseNet-121 | Supervised (classification) | -------- |
| VGG^24^ | VGG-11 | Supervised (classification) | -------- |
| VGG^24^ | VGG-13 | Supervised (classification) | -------- |
| VGG^24^ | VGG-16 | Supervised (classification) | -------- |
| VGG^24^ | VGG-19 | Supervised (classification) | -------- |
| MobileNet^25^ | MobileNet | Supervised (classification) | -------- |
| SqueezeNet^26^ | SqueezeNet-10 | Supervised (classification) | -------- |
| SqueezeNet^26^ | SqueezeNet-11 | Supervised (classification) | -------- |
| ResNet^27^ | ResNext-50 | Supervised (classification) | -------- |
| ResNet^27^ | ResNet-101 | Supervised (classification) | -------- |
| MnasNet^28^ | MnasNet_05 | Supervised (classification) | -------- |
| MnasNet^28^ | MnasNet_10 | Supervised (classification) | -------- |
| ShuffleNet^29^ | ShuffleNet_05 | Supervised (classification) | -------- |
| ShuffleNet^29^ | ShuffleNet_10 | Supervised (classification) | -------- |

**Supplementary file 1b. Models tested.** For each model, we measured representational factorization and invariance in each of the final five layers of the model as well as evaluating their brainlikeness using the datasets in **Supplementary file 1b.**

## REFERENCES

1. Majaj, N. J., Hong, H., Solomon, E. A. & DiCarlo, J. J. Simple Learned Weighted Sums of Inferior Temporal Neuronal Firing Rates Accurately Predict Human Core Object Recognition Performance. *J. Neurosci.* **35**, 13402–13418 (2015).

2. Rust, N. C. & DiCarlo, J. J. Balanced Increases in Selectivity and Tolerance Produce Constant Sparseness along the Ventral Visual Stream. *J. Neurosci.* **32**, 10170–10182 (2012).

3. Rajalingham, R. *et al.* Large-Scale, High-Resolution Comparison of the Core Visual Object Recognition Behavior of Humans, Monkeys, and State-of-the-Art Deep Artificial Neural Networks. *J. Neurosci.* **38**, 7255–7269 (2018).

4. Kay, K. N., Naselaris, T., Prenger, R. J. & Gallant, J. L. Identifying natural images from human brain activity. *Nature* **452**, 352–355 (2008).

5. Shen, G., Horikawa, T., Majima, K. & Kamitani, Y. Deep image reconstruction from human brain activity. *PLOS Comput. Biol.* **15**, e1006633 (2019).

6. Chen, T., Kornblith, S., Norouzi, M. & Hinton, G. A Simple Framework for Contrastive Learning of Visual Representations. *ArXiv200205709 Cs Stat* (2020).

7. He, K., Fan, H., Wu, Y., Xie, S. & Girshick, R. Momentum Contrast for Unsupervised Visual Representation Learning. *ArXiv191105722 Cs* (2020).

8. Chen, X., Fan, H., Girshick, R. & He, K. Improved Baselines with Momentum Contrastive Learning. *ArXiv200304297 Cs* (2020).

9. Wu, Z., Xiong, Y., Yu, S. & Lin, D. Unsupervised Feature Learning via Non-Parametric Instance-level Discrimination. *ArXiv180501978 Cs* (2018).

10. Tian, Y. *et al.* What makes for good views for contrastive learning. *ArXiv200510243 Cs* (2020).

11. Caron, M. *et al.* Unsupervised Learning of Visual Features by Contrasting Cluster Assignments. *ArXiv200609882 Cs* (2020).

12. Caron, M., Bojanowski, P., Joulin, A. & Douze, M. Deep Clustering for Unsupervised Learning of Visual Features. *ArXiv180705520 Cs* (2019).

13. Grill, J.-B. *et al.* Bootstrap Your Own Latent: A New Approach to Self-Supervised Learning. *ArXiv200607733 Cs Stat* (2020).

14. Gidaris, S., Bursuc, A., Komodakis, N., Pérez, P. & Cord, M. Boosting Few-Shot Visual Learning with Self-Supervision. Preprint at https://doi.org/10.48550/arXiv.1906.05186 (2019).

15. Zhang, R., Isola, P. & Efros, A. A. Colorful Image Colorization. Preprint at https://doi.org/10.48550/arXiv.1603.08511 (2016).

16. Noroozi, M. & Favaro, P. Unsupervised Learning of Visual Representations by Solving Jigsaw Puzzles. Preprint at https://doi.org/10.48550/arXiv.1603.09246 (2017).

17. Donahue, J. & Simonyan, K. Large Scale Adversarial Representation Learning. in *Advances in Neural Information Processing Systems* vol. 32 (Curran Associates, Inc., 2019).

18. He, K., Zhang, X., Ren, S. & Sun, J. Deep Residual Learning for Image Recognition. *ArXiv151203385 Cs* (2015).

19. Zagoruyko, S. & Komodakis, N. Wide Residual Networks. Preprint at https://doi.org/10.48550/arXiv.1605.07146 (2017).

20. Krizhevsky, A., Sutskever, I. & Hinton, G. E. ImageNet Classification with Deep Convolutional Neural Networks. in *Advances in Neural Information Processing Systems 25* (eds. Pereira, F., Burges, C. J. C., Bottou, L. & Weinberger, K. Q.) 1097–1105 (Curran Associates, Inc., 2012).

21. Szegedy, C. *et al.* Going Deeper with Convolutions. *ArXiv14094842 Cs* (2014).

22. Szegedy, C., Vanhoucke, V., Ioffe, S., Shlens, J. & Wojna, Z. Rethinking the Inception Architecture for Computer Vision. Preprint at https://doi.org/10.48550/arXiv.1512.00567 (2015).

23. Huang, G., Liu, Z., van der Maaten, L. & Weinberger, K. Q. Densely Connected Convolutional Networks. Preprint at https://doi.org/10.48550/arXiv.1608.06993 (2018).

24. Simonyan, K. & Zisserman, A. Very Deep Convolutional Networks for Large-Scale Image Recognition. Preprint at https://doi.org/10.48550/arXiv.1409.1556 (2015).

25. Howard, A. G. *et al.* MobileNets: Efficient Convolutional Neural Networks for Mobile Vision Applications. Preprint at https://doi.org/10.48550/arXiv.1704.04861 (2017).

26. Iandola, F. N. *et al.* SqueezeNet: AlexNet-level accuracy with 50x fewer parameters and <0.5MB model size. Preprint at https://doi.org/10.48550/arXiv.1602.07360 (2016).

27. Xie, S., Girshick, R., Dollár, P., Tu, Z. & He, K. Aggregated Residual Transformations for Deep Neural Networks. *ArXiv161105431 Cs* (2017).

28. Tan, M. *et al.* MnasNet: Platform-Aware Neural Architecture Search for Mobile. Preprint at https://doi.org/10.48550/arXiv.1807.11626 (2019).

29. Zhang, X., Zhou, X., Lin, M. & Sun, J. ShuffleNet: An Extremely Efficient Convolutional Neural Network for Mobile Devices. Preprint at https://doi.org/10.48550/arXiv.1707.01083 (2017).
